# ﻿Diversity, conservation, and endemism of the bats (Chiroptera) of Mexico

**DOI:** 10.3897/zookeys.1261.163471

**Published:** 2025-11-21

**Authors:** Ameyalli Marín-Ventura, Liliana Rosas-Durán, Cárol Sierra-Durán, Joaquín Arroyo-Cabrales, Rodrigo A. Medellín

**Affiliations:** 1 Laboratorio de Ecología y Conservación de Vertebrados Terrestres, Instituto de Ecología, Universidad Nacional Autónoma de México, Coyoacán, Mexico City C.P 04510, Mexico Universidad Nacional Autónoma de México Mexico City Mexico; 2 BiBio Research Group, Natural Sciences Museum of Granollers, Av/Francesc Macià 51, Granollers 08402, Spain BiBio Research Group, Natural Sciences Museum of Granollers Granollers Spain; 3 Laboratorio de Arqueozoología, Subdirección de Laboratorios y Apoyo Académico, Instituto Nacional de Antropología e Historia, Mexico City, Mexico Subdirección de Laboratorios y Apoyo Académico, Instituto Nacional de Antropología e Historia Mexico City Mexico

**Keywords:** Hibernation, Latin America, migration, richness, species list, taxonomic update

## Abstract

An updated checklist of the bats of Mexico is presented, comprising eight families, 71 genera, and 146 species. Since the last checklist published by Ramírez-Pulido et al. (2014), two new species have been described: *Vampyressa villai* and *Corynorhinus leonpaniaguae*, and two more, *Phyllops falcatus* and *Phyllostomus hastatus*, were recorded for the first time in the country. Additionally, 22 taxonomic changes have occurred since the previous list. Of the 146 bat species recorded, 20 are endemic to Mexico, highlighting the country’s megadiversity, reflection of its unique biogeographic and ecological characteristics. This remarkable distinction also brings significant responsibility, as Mexico holds the highest number of endemic bat species worldwide. Consequently, protecting these endemic species and those at risk of extinction must be a top priority in the country’s decision-making processes and conservation policies.

## ﻿Introduction

Because of its complex geography, which includes extensive mountain ranges such as the Sierra Madre Oriental, the Sierra Madre Occidental, and the Trans-Mexican Neovolcanic Belt, as well as tropical jungles, deserts, and coastal ecosystems, and also because its longitudinal extent from 14 to 32 degrees north, encompassing the complete limits between the Nearctic and the Neotropical regions around the tropic of Cancer, Mexico is one of the most biodiverse countries in the world (Morrone 2019). The contrasting topography and climatic conditions of Mexico have fostered this remarkable biological richness, with bat diversity standing out among mammalian groups (Medellín et al. 2008).

Bats (Chiroptera) represent the second-largest group of mammals in terms of species richness in Mexico and worldwide (Simmons and Cirranello 2024). With over 140 species recorded in the country, bats play essential roles in ecosystems by participating in key processes such as plant pollination, insect population control, and seed dispersal (Medellín et al. 2017). Due to their sensitivity to environmental changes, bats also serve as excellent indicators of the conservation status of ecosystems, ranging from temperate forests to urban and tropical environments (Medellín et al. 2000; Basile et al. 2020; Ramos-H et al. 2020; Cajaiba et al. 2021; Dambly et al. 2021; Gutiérrez-Granados and Rodríguez-Zúñiga 2024).

Very little has been done on two ecology strategies of Mexican bats: migration and hibernation. Migration is defined as a cyclical, seasonal, predictable, and synchronized movement between two regions, usually separated by long distances, and enabling species to complete distinct stages of their life cycle and optimize adaptation to seasonally available resources (Burke et al. 2019). Hibernation is a controlled reduction of body temperature and physiological functions that can last days, weeks, or months and requires prior fat accumulation (Altringham 2011; Geiser 2021). Understanding these strategies and which species of Mexican bats employ them is essential for guiding conservation efforts.

Despite their importance, information regarding the ecology and distribution of many bat species remains limited. Most research efforts focus on recovery programs or are targeted toward specific species. That is the case of *Leptonycteris yerbabuenae* Martínez & Villa-R, 1940, a species that was considered Threatened in Mexico and Endangered in the U.S. This attracted much attention and lots of research prompted its recovery by 2013 (Medellín and Torres-Knoop 2015; Medellín et al. 2018; Medellín 2019; Laverty et al. 2025). Other species or aspects of the biology of bats have also produced focal work on particular topics such as molecular genetics, evolution, ecology, or conservation.

The study of these mammals is an evolving field, and in recent years, the list of species in Mexico and the rest of the world has increased significantly due to new taxonomic and biogeographic research. Mexico has witnessed a recent strong impulse studying the taxonomy and distribution of bats. As a result, new species and previously unrecorded species have been recently discovered in the country (e.g. Aranda-Coello et al. 2025; López-Cuamatzi et al. 2024). Given this dynamism, maintaining an updated list of bats in Mexico is crucial for assessing their conservation status, understanding their diversity patterns, and designing effective protection strategies and policies (Simmons and Cirranello 2024).

In this context, it is essential to gather and analyze the most recent knowledge about the bat species present in Mexican territory, considering their levels of endemism and their national and international conservation status. This update will not only strengthen scientific research but will also provide key information for decision-making regarding conservation and the management of the ecosystems in which these organisms play vital roles.

## ﻿Methodology

This list compiles all species currently recognized in the literature by most authors as resident in Mexico. Through a comprehensive analysis, we identified species whose taxonomy or distribution has changed and incorporated recently described species with a distribution in the country. We also assessed their conservation status at both national and international levels, as well as their geographic distribution, to identify endemic species and areas with greater species richness within Mexican territory.

To compile this list, we conducted a critical assessment to include each species that have been recently recognized or newly reported in the country. Our work was based on the national lists by Medellín et al. (2008) and Ramírez-Pulido et al. (2014), as well as the international lists by Wilson and Mittermeier (2019) and Simmons and Cirranello (2024). Additionally, we incorporated taxonomic revisions published after the listing by Ramírez-Pulido et al. (2014) such as Rivas-Camo et al. (2020), Garbino et al. (2024), and López-Cuamatzi et al. (2024). It is necessary to mention that we are not following the ASM Mammal Diversity Database because there is a lag between the new taxonomic proposals and their appearance in such a database (Mammal Diversity Database 2025).

The geographic distribution of species was determined by reviewing the distribution maps provided by Wilson and Mittermeier (2019), Simmons and Cirranello (2024), databases from Comisión Nacional para el Conocimiento y Uso de la Biodiversidad (CONABIO), Global Biodiversity Information Facility (GBIF), and International Union for Conservation of Nature (IUCN), as well as previous studies on the presence of bats in different regions of Mexico such as the bats of Tabasco (García-Morales, 2021; Pérez-Netzahual et al. 2023), Campeche (Mejenes-López et al. 2024), Veracruz (Cerón-Hernández et al. 2022), and Durango (Torres-Morales et al. 2010), among others. Based on their distribution, we identified species endemic to Mexico.

The conservation status of each species included in the list was determined using the classification from the most recent version of the Mexican federal list of endangered species (NOM-059-SEMARNAT-2025) and the IUCN Red List. For species evaluated in the Mexican federal list, we used the categories “Threatened”, “Special Protection”, and “Endangered”. Meanwhile, for species assessed in the IUCN Red List, we considered the categories “Near Threatened”, “Vulnerable”, “Endangered”, and “Critically Endangered” to identify those requiring urgent conservation attention.

## ﻿Results

A total of 146 bat species are recognized in Mexico, classified into 71 genera and eight families (Table 1). This places the country among the top ten worldwide in bat diversity. As a result, the list of bat species of Mexico has expanded compared to the last update by Ramírez-Pulido et al. (2014), which recorded 139 species.

**Table 1.** Full list of the bat species of Mexico.

Family/Subfamily	Genus	Species
** Emballonuridae **	* Balantiopteryx *	*Balantiopteryx io* Thomas, 1904
		*Balantiopteryx plicata* Peters, 1867
	* Centronycteris *	*Centronycteris centralis* Thomas, 1912
	* Diclidurus *	*Diclidurus albus* Wied-Neuwied, 1820
	* Peropteryx *	*Peropteryx kappleri* Peters, 1867
		*Peropteryx macrotis* (J. A. Wagner, 1820)
	* Rhynchonycteris *	*Rhynchonycteris naso* (Wied-Neuwied, 1820)
	* Saccopteryx *	*Saccopteryx bilineata* (Temminck, 1838)
		*Saccopteryx leptura* (Schreber, 1774)
** Noctilionidae **	* Noctilio *	*Noctilio albiventris* Desmarest, 1818
		*Noctilio leporinus* (Linnaeus, 1758)
** Thyropteridae **	* Thyroptera *	*Thyroptera tricolor* Spix, 1823
** Mormoopidae **	* Mormoops *	*Mormoops megalophylla* (Peters, 1864)
	* Pteronotus *	*Pteronotus fulvus* (Thomas, 18)
		*Pteronotus gymnonotus* (J. A. Wagner, 1843)
		*Pteronotus mesoamericanus* (Smith, 1972)
		*Pteronotus mexicanus* (Miller, 1902)
		*Pteronotus psilotis* (Dobson, 1878)
** Phyllostomidae **		
** Macrotinae **	* Macrotus *	*Macrotus californicus* Baird, 1858
		*Macrotus waterhousii* Gray, 1843
** Micronycterinae **	* Lampronycteris *	*Lampronycteris brachyotis* (Dobson, 1879)
	* Micronycteris *	*Micronycteris microtis* Miller, 1898
		*Micronycteris schmidtorum* Sanborn, 1935
	* Trinycteris *	*Trinycteris nicefori* (Sanborn, 1949)
** Desmodontinae **	* Desmodus *	*Desmodus rotundus* (É. Geoffroy Saint-Hilaire, 1810)
	* Diaemus *	*Diaemus youngi* (Jentink, 1893)
	* Diphylla *	*Diphylla ecaudata* Spix, 1823
** Lonchorhininae **	* Lonchorhina *	*Lonchorhina aurita* Tomes, 1863
** Phyllostominae **	* Chrotopterus *	*Chrotopterus auritus* (Peters, 1856)
	* Gardnerycteris *	*Gardnerycteris keenani* (Handley, 1960)
	* Lophostoma *	*Lophostoma nicaraguae* (Goodwin, 1942)
		*Lophostoma evotis* (W. B. Davis & Carter, 1978)
	* Macrophyllum *	*Macrophyllum macrophyllum* (Schinz, 1821)
	* Mimon *	*Mimon cozumelae* Goldman 1914
	* Phylloderma *	*Phylloderma stenops* (Peters, 1865)
	* Phyllops *	*Phyllops falcatus* (Gray, 1839)
	* Phyllostomus *	*Phyllostomus discolor* (Wagner, 1843)
		*Phyllostomus hastatus* (Pallas, 1767)
	* Tonatia *	*Tonatia bakeri* Williams, Willig & Reid, 1995
	* Trachops *	*Trachops coffini* Goldman, 1925
	* Vampyrum *	*Vampyrum spectrum* (Linnaeus, 1758)
** Glossophaginae **	* Anoura *	*Anoura peruana lasiopyga* (Peters, 1868)
	* Choeroniscus *	*Choeroniscus godmani* (Thomas, 1903)
	* Choeronycteris *	*Choeronycteris mexicana* Tschudi, 1844
	* Glossophaga *	*Glossophaga commissarisi* Gardner, 1962
		*Glossophaga leachii* (Gray, 1844)
		*Glossophaga morenoi* Martínez & Villa-R., 1938
		*Glossophaga mutica* Merriam, 1898
	* Hylonycteris *	*Hylonycteris underwoodi* Thomas, 1903
	* Leptonycteris *	*Leptonycteris nivalis* (Saussure, 1860)
		*Leptonycteris yerbabuenae* Martínez & Villa-R, 1940
	* Lichonycteris *	*Lichonycteris obscura* Thomas, 1895
	* Musonycteris *	*Musonycteris harrisoni* Schaldach & McLaughlin, 1960
** Carollinae **	* Carollia *	*Carollia perspicillata* (Linnaeus, 1758)
		*Carollia sowelli* Baker, Solari & Hoffmann, 2002
		*Carollia subrufa* (Hahn, 1905)
** Glyphonycterinae **	* Glyphonycteris *	*Glyphonycteris sylvestris* Thomas, 1896
** Stenodermatinae **	* Artibeus *	*Artibeus hirsutus* K. Andersen, 1906
		*Artibeus intermedius* J.A. Allen, 1897
		*Artibeus jamaicensis* Leach, 1821
		*Artibeus lituratus* (Olfers, 1818)
	* Centurio *	*Centurio senex* Gray, 1842
	* Chiroderma *	*Chiroderma salvini* Dobson, 1878
		*Chiroderma scopaeum* Handley, 1966
		*Chiroderma villosum* Peters, 1860
	* Dermanura *	*Dermanura azteca* (K. Andersen, 1906)
		*Dermanura phaeotis* Miller, 1902
		*Dermanura tolteca* (Saussure, 1860)
		*Dermanura watsoni* (Thomas, 1901)
	* Enchisthenes *	*Enchisthenes hartii* (Thomas, 1892)
	* Platyrrhinus *	*Platyrrhinus helleri* (Peters, 1866)
	* Sturnira *	*Sturnira hondurensis* Goodwin, 1940
		*Sturnira parvidens* Goldman, 1917
	* Uroderma *	*Uroderma convexum* Lyon, 1902
		*Uroderma davisi* Baker & McDaniel, 1972
		*Uroderma magnirostrum* Davis, 1968
	* Vampyressa *	*Vampyressa thyone* Thomas, 1909
		*Vampyressa villai* Garbino, Hernández-Canchola, León-Paniagua & Tavares, 2024
	* Vampyrodes *	*Vampyrodes major* Allen, 1908
** Natalidae **	* Natalus *	*Natalus mexicanus* Miller, 1902
		*Natalus lanatus* Tejedor, 2005
** Molossidae **	* Cynomops *	*Cynomops mexicanus* (Jones & Genoways, 1967)
	* Eumops *	*Eumops auripendulus* (Shaw, 1800)
		*Eumops ferox* (Gundlach in Peters, 1861)
		*Eumops hansae* Sanborn, 1932
		*Eumops nanus* (Miller, 1900)
		*Eumops perotis* (Schinz, 1821)
		*Eumops underwoodi* Goodwin, 1940
	* Molossus *	*Molossus alvarezi* Gonzalez-Ruiz, Ramirez-Pulido & Arroyo-Cabrales, 2011
		*Molossus aztecus* Saussure, 1860
		*Molossus coibensis* J. A. Allen, 1904
		*Molossus molossus* (Pallas, 1766)
		*Molossus nigricans* Miller, 1902
		*Molossus sinaloae* J. A. Allen, 1906
	* Nyctinomops *	*Nyctinomops aurispinosus* (Peale, 1849)
		*Nyctinomops femorosaccus* (Merriam, 1889)
		*Nyctinomops laticaudatus* (É. Geoffroy Saint-Hilaire, 1805)
		*Nyctinomops macrotis* (Gray, 1839)
	* Promops *	*Promops centralis* Thomas, 1915
	* Tadarida *	*Tadarida brasiliensis* (I. Geoffroy Saint-Hilaire, 1824)
** Vespertilionidae **	* Antrozous *	*Antrozous pallidus* (Le Conte, 1856)
	* Baeodon *	*Baeodon alleni* (Thomas, 1892)
		*Baeodon gracilis* (Miller, 1897)
	* Bauerus *	*Bauerus dubiaquercus* (Van Gelder, 1959)
	* Corynorhinus *	*Corynorhinus leonpaniaguae* López-Cuamatzi, Ortega, Ospina-Garcés, Zúñiga & MacSwiney G., 2024
		*Corynorhinus mexicanus* G. M. Allen, 1916
		*Corynorhinus townsendii* (Cooper, 1837)
	* Neoeptesicus *	*Neoeptesicus brasiliensis* (Desmarest, 1819)
		*Neoeptesicus furinalis* (d’Orbigny & Gervais, 1847)
	* Eptesicus *	*Eptesicus fuscus* (Palisot de Beauvois, 1796)
	* Euderma *	*Euderma maculatum* (J. A. Allen, 1891)
	* Idionycteris *	*Idionycteris phyllotis* (G. M. Allen, 1916)
	* Lasionycteris *	*Lasionycteris noctivagans* (Le Conte, 1831)
	* Lasiurus *	*Lasiurus borealis* (Müller, 1776)
		*Lasiurus cinereus* (Palisot de Beauvois, 1796)
		*Lasiurus ega* (Gervais, 1856)
		*Lasiurus frantzii* (Peters, 1870)
		*Lasiurus intermedius* H. Allen, 1862
		*Lasiurus xantinus* (Thomas, 1897)
	* Myotis *	*Myotis albescens* (È. Geoffroy Saint-Hilaire, 1806)
		*Myotis auriculus* Baker & Stains, 1955
		*Myotis californicus* (Audubon & Bachman, 1842)
		*Myotis carteri* (LaVal, 1973)
		*Myotis elegans* Hall, 1962
		*Myotis evotis* (H. Allen, 1864)
		*Myotis extremus* Miller & Allen, 1928
		*Myotis findleyi* Bogan, 1978
		*Myotis fortidens* Miller & Allen, 1928
		*Myotis ciliolabrum* (Merriam, 1886)
		*Myotis occultus* Hollister, 1909
		*Myotis pilosatibialis* LaVal, 1973
		*Myotis planiceps* R.H. Baker, 1955
		*Myotis thysanodes* Miller, 1897
		*Myotis velifer* (J. A. Allen, 1890)
		*Myotis vivesi* Menegaux, 1901
		*Myotis volans* (H. Allen, 1866)
		*Myotis yumanensis* (H. Allen, 1866)
	* Nycticeius *	*Nycticeius humeralis* (Rafinesque, 1818)
	* Parastrellus *	*Parastrellus hesperus* (H. Allen, 1864)
	* Perimyotis *	*Perimyotis subflavus* (F. Cuvier, 1832)
	* Rhogeessa *	*Rhogeessa aenea* Goodwin, 1958
		*Rhogeessa bickhami* Baird, Marchan-Rivadeneira, Perez & Baker, 2012
		*Rhogeessa genowaysi* Baker, 1984
		*Rhogeessa mira* LaVal, 1973
		*Rhogeessa parvula* H. Allen, 1866
		*Rhogeessa tumida* H. Allen, 1866

This increase is largely attributed to significant advances in molecular research over the past decade, which have enabled a more comprehensive understanding of bat diversity. These developments have facilitated the taxonomic revision and reclassification of several species, as well as the description of newly recognized taxa, such as *Vampyressa villai* Garbino, Hernández-Canchola, León-Paniagua & Tavares, 2024 and *Corynorhinus leonpaniaguae* López-Cuamatzi, Ortega, Ospina-Garcés, Zúñiga & MacSwiney G., 2024. Both descriptions were supported by integrative studies combining detailed morphological analyses, including cranial and dental traits, with genetic evidence based on mitochondrial and nuclear DNA sequences (Garbino et al. 2024; López-Cuamatzi et al. 2024).

Additionally, the presence of *Phyllops falcatus* (Gray, 1839) a species not previously recognized as part of Mexico’s bat fauna, has been documented. This Caribbean-endemic bat was recently recorded on Cozumel Island, where resident populations have been confirmed (Rivas-Camo et al. 2020), thus significantly extending the known geographic range of the genus. Its recent discovery in the country suggests possible previously undocumented dispersal routes or shifts in distribution patterns, indicating that additional populations in Mexico may yet be found.

Likewise, *Phyllostomus hastatus* (Pallas, 1767) was recently reported in Mexico (Aranda-Coello et al. 2025). This record extends the species’ northern distribution by approximately 120 km from the nearest previously documented occurrence in Guatemala and represents the second species of the genus recorded in our country.

On the other hand, the presence of *Natalus lanatus* Tejedor, 2005 is recognized as the second species of the genus *Natalus* in Mexico, supported by previous studies (Rodríguez-Herrera et al. 2011; Ramírez-Pulido et al. 2014; Tejedor 2019), which reinforces the need to continue evaluating cryptic diversity in the country.

### ﻿Species with changes in nomenclature and taxonomy

Taxonomic analyses of the chiropteran fauna have identified 23 bat species in Mexico that have undergone taxonomic restructuring and nomenclatural changes, spanning four families: Mormoopidae, Phyllostomidae, Molossidae, and Vespertilionidae (Table 2). Notably, several genera previously thought to contain a limited or poorly defined number of species have been shown to encompass greater diversity, including taxa endemic to Mexico. These revisions include the redefinition of geographic ranges for species once considered widespread and the formal description of new species, supported by morphological, genetic, and ecological evidence. In all cases, the Mexican taxa maintain close affinities with the original species complexes but exhibit sufficient divergence to justify their recognition as distinct species.

**Table 2.** Taxonomic changes in bats of Mexico from 2014 to 2024. Updates for a total of 22 species were recorded distributed across four families.

Family	Current nomenclature	Previous nomenclature	Bibliographic reference
** Mormoopidae **	*Pteronotus fulvus*	*Pteronotus davyi*	Pavan and Marroig 2016
	*Pteronotus mesoamericanus*	*Pteronotus parnellii*	Pavan and Marroig 2016
	*Pteronotus mexicanus*	*Pteronotus parnellii mexicanus*	Pavan and Marroig 2016
	*Pteronotus psilotis*	*Pteronotus personatus*	Pavan and Marroig 2016
** Phyllostomidae **	*Anoura peruana lasiopyga*	*Anoura geoffroyi*	Molinari et al. 2023
	*Artibeus intermedius*	*Artibeus lituratus intermedius*	Larsen et al. 2013
	*Chiroderma scopaeum*	*Chiroderma salvini scopaeum*	Garbino et al. 2020
	*Glossophaga mutica*	*Glossophaga soricina mutica*	Calahorra-Oliart et al. 2021
	*Gardnerycteris keenani*	*Mimon crenulatum*	Hurtado and D’Elía 2018
	*Lophostoma nicaraguae*	*Lophostoma brasiliense*	Esquivel et al. 2022
	*Tonatia bakeri*	*Tonatia saurophila*	Basantes et al. 2020
	*Trachops coffni*	*Trachops cirrhosus*	Fonseca et al. 2024
	*Uroderma convexum*	*Uroderma bilobatum convexum*	Mantilla-Meluk 2014
	*Uroderma davisi*	*Uroderma bilobatum*	Mantilla-Meluk 2014
** Molossidae **	*Molossus nigricans*	*Molossus rufus*	Loureiro et al. 2020
** Vespertilionidae **	*Baeodon alleni*	*Rhogeessa alleni*	Roehrs et al. 2011
	*Baeodon gracilis*	*Rhogeessa gracilis*	Roehrs et al. 2011
	*Lasiurus frantzii*	*Lasiurus blossevillii*	Baird et al. 2015
	*Myotis carteri*	*Myotis nigricans carteri*	Novaes et al. 2022
	*Myotis extremus*	*Myotis nigricans extremus*	Novaes et al. 2024
	*Myotis pilosatibialis*	*Myotis keaysi*	Carrion-Bonilla and Cook 2020
	*Neoeptesicus brasiliensis*	*Eptesicus brasiliensis*	Cláudio et al. 2023
	*Neoeptesicus furinalis*	*Eptesicus furinalis*	Cláudio et al. 2023

Moreover, several taxa previously classified as subspecies have been reclassified as independent species, thereby redefining their taxonomic rank and improving the resolution of bat biodiversity assessments in Mexico. Among the most notable nomenclatural changes is *Anoura peruana lasiopyga* (Peters, 1868; formerly *A. geoffroyi*), whose recognition as a distinct species is supported by morphometric analyses, ecological niche modeling, and DNA barcoding (Molinari et al. 2023). Similarly, phylogenetic and morphological analyses by Fonseca et al. (2024) revealed that the genus *Trachops* comprises at least three distinct species, with *Trachops coffini* Goldman, 1925 (previously *T. cirrhosus*) being the only one occurring in Mexico.

In addition, *Artibeus intermedius* J.A. Allen, 1897, a species not previously included in earlier checklists, is recognized here based on morphological and molecular evidence provided by Marchán-Rivadeneira et al. (2012) and Larsen et al. (2013), respectively. These studies highlighted ecological differences between *A. intermedius* and closely related species as a key factor contributing to the speciation process.

The family Vespertilionidae has undergone significant taxonomic revisions. The genus *Baeodon* was initially separated from *Rhogeessa* in the 1970s and 1980s, though it was later considered a subgenus. However, Roehrs et al. (2011), through nuclear DNA analyses, confirmed that *Baeodon* is a distinct genus comprising two species, *Baeodon alleni* (Thomas, 1892) and *Baeodon gracilis* (Miller, 1897), both of which are endemic to Mexico. In addition, a new genus, *Neoeptesicus*, was recently established to accommodate *Neoeptesicus brasiliensis* (Desmarest, 1819) and *Neoeptesicus furinalis* (d’Orbigny & Gervais, 1847), species previously included in the genus *Eptesicus* (Cláudio et al. 2023).

### ﻿Bat richness patterns in Mexico

**Figure 1.**
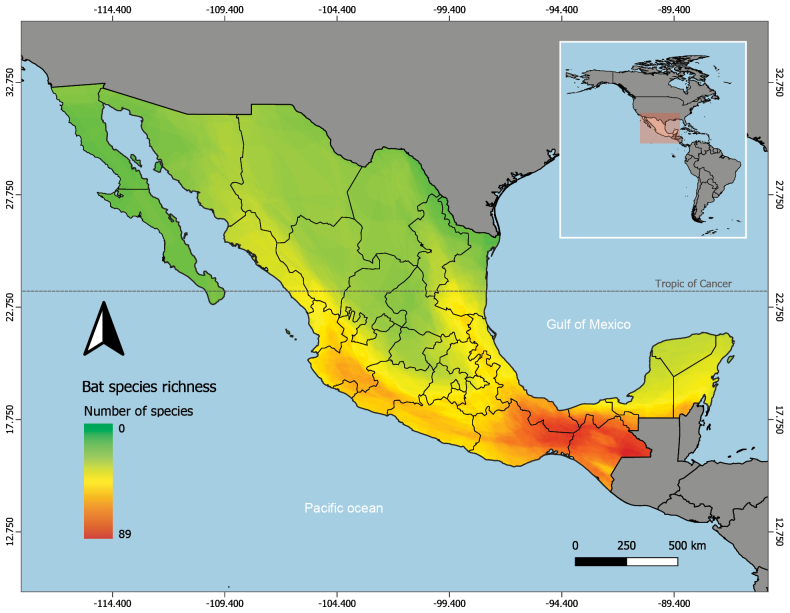
Gradient of bat species richness in Mexico. Species diversity is higher in the southern part of the country, where tropical habitats predominate, and decreases toward the north in more arid areas. In this representation, red indicates a greater number of species, while green represents lower species richness.

The bat fauna of Mexico faithfully reflects the country’s megadiversity, as well as its biogeographic and ecological patterns and processes. Bat species richness follows a distinct latitudinal gradient (Fig. 1). Southern regions, particularly in the states of Chiapas, Oaxaca, Veracruz, and the Yucatán Peninsula, show the highest species richness, with up to 89 species recorded in the Selva Lacandona region of Chiapas, Mexico precisely what would be expected from deep Neotropical affinities. In contrast, northern areas and the central highlands display lower diversity, with significantly fewer species, as would correspondingly be expected for the Nearctic region. As is well known, Mexico is the only country that contains the entirety of the limits between any two major Biogeographical regions, so the northern half of Mexico is part of the Nearctic region, and the southern half is part of the Neotropical region. As with other taxonomic groups such as reptiles, amphibians, and plants, the highest bat diversity occurs in the Neotropical region of southern Mexico. This pattern reflects the greater ecological complexity of tropical environments, with diversity decreasing progressively toward the north as conditions become increasingly arid and temperate.

### ﻿Endemism of bats in Mexico

Twenty bat species endemic to Mexico were identified, spanning 12 genera and four families (Table 3). The family Vespertilionidae is particularly notable for its high diversity, accounting for over 60% of the endemic species recorded. These results underscore Mexico’s exceptional richness and uniqueness in bat biodiversity, establishing the country as both a continental leader in endemic bat species and a critical center of chiropteran endemism.

**Table 3.** Endemic species of Mexico, including national (NOM-059-SEMARNAT, 2025), and international (IUCN) risk categories. IUCN conservation status is Least Concern unless indicated otherwise.

Family	Genus	Species	Conservation status NOM-059-SEMARNAT-2025	Conservation status IUCN
** Mormoopidae **	* Pteronotus *	*P. mexicanus*		
** Phyllostomidae **	* Artibeus *	*A. hirsutus*		
	* Chiroderma *	*C. scopaeum*		
	* Glossophaga *	*G. morenoi*		
	* Musonycteris *	*M. harrisoni*	P	VU
	* Vampyressa *	*V. villai*		
** Molossidae **	* Cynomops *	*C. mexicanus*	Pr	
	* Molossus *	*M. sinaloae*		
** Vespertilionidae **	* Baeodon *	*B. alleni*		
		*B. gracilis*		
	* Corynorhinus *	*C. leonpaniaguae*		
		*C. mexicanus*		NT
	* Myotis *	*M. carteri*	Pr	
		*M. findleyi*		EN
		*M. planiceps*	P	EN
		*M. vivesi*	P	VU
	* Rhogeessa *	*R. genowaysi*	A	EN
		*R. mira*	Pr	VU
		*R. parvula*		
		*R. tumida*		

The risk category is indicated according to NOM-059-SEMARNAT-2025: Subject to Special Protection = Pr; Threatened = A; Endangered = P. The risk categories according to the IUCN are also provided: Near Threatened = NT; Vulnerable = VU; Endangered = EN.

On the other hand, 45% of the Mexican endemic bat species fall under some risk category, either in NOM-059-SEMARNAT-2025 or the IUCN Red List. Among these, five species are in a critical condition, as they are listed as threatened under both regulatory frameworks: *Musonycteris harrisoni* Schaldach & McLaughlin, 1960, *Myotis planiceps* R.H. Baker, 1955, *Myotis vivesi* Menegaux, 1901, *Rhogeessa genowaysi* R.J. Baker, 1984, and *Rhogeessa mira* LaVal, 1973. In particular, those classified as Endangered (EN) by the IUCN face a high risk of extinction in the short term.

Most Mexican endemic species are located in the tropical dry forests of the western and southern regions of the country, within the Mexican Transition Zone (**MTZ**), and are considered a high conservation priority due to their extraordinary biological richness and the increasing levels of threat and deforestation (Escalante et al. 2009, 2013). Information on their conservation status underscores the urgency of strengthening monitoring, management, and protection efforts in these key areas for Mexico’s endemic biodiversity.

### ﻿Conservation status

The most recent version of Mexico’s federal endangered species list, NOM-059-SEMARNAT-2025, which was recently released for public consultation (SEMARNAT 2025), includes 38 bat species categorized under three levels of risk: 17 under Special Protection (Pr), 16 as Threatened (A), and five as Endangered (P). Notably, this update proposes the removal of the lesser long-nosed bat (*Leptonycteris yerbabuenae*) from the list (Medellín and Torres-Knoop 2015), reflecting the success of conservation actions implemented for this species in Mexico. In contrast, the IUCN Red List recognizes 14 bat species from Mexico under threat categories: five as Vulnerable (VU), five as Near Threatened (NT), and four as Endangered (EN). These differences highlight the distinct evaluation criteria used by each system (Table 4).

**Table 4.** Conservation statuses and categories of bats in Mexico according to the global system of the International Union for Conservation of Nature (IUCN) Red List, the national system of the Norma Oficial Mexicana 059 (NOM-59; Mexican Official Standard-059), and expert elicitation by Adams et al. (2024).

Family/Subfamily	Genus	Species	Conservation status NOM-059-SEMARNAT-2025	Conservation status IUCN	Adams et al. 2024
** Emballonuridae **	* Balantiopteryx *	*B. io*	NL	VU	
	* Centronycteris *	*C. centralis*	Pr	LC	
	* Peropteryx *	*P. kappleri*	Pr	LC	
	* Rhynchonycteris *	*R. naso*	Pr	LC	
	* Saccopteryx *	*S. leptura*	Pr	LC	Imperiled
** Noctilionidae **	* Noctilio *	*N. albiventris*	Pr	LC	
** Thyropteridae **	* Thyroptera *	*T. tricolor*	Pr	LC	
** Mormoopidae **	* Pteronotus *	*P. gymnonotus*	A	LC	
** Phyllostomidae **					
** Desmodontinae **	* Diaemus *	*D. youngi*	Pr	LC	
** Glossophaginae **	* Choeronycteris *	*C. mexicana*	A	NT	
	* Leptonycteris *	*L. nivalis*	A	EN	
		*L. yerbabuenae*	Recovered	NT	
	* Lichonycteris *	*L. obscura*	NL	LC	Imperiled
	* Musonycteris *	*M. harrisoni*	P	VU	Critically Imperiled
** Lonchorhininae **	* Lonchorhina *	*L. aurita*	A	LC	
** Micronycterinae **	* Lampronycteris *	*L. brachyotis*	A	LC	
	* Micronycteris *	*M. schmidtorum*	A	LC	
	* Trinycteris *	*T. nicefori*	NL	LC	Imperiled
** Phyllostominae **	* Chrotopterus *	*C. auritus*	A	LC	
	* Gardnerycteris *	*G. keenani*	A*	LC	
	* Lophostoma *	*L. nicaraguae*	A*	LC	
		*L. evotis*	A	LC	
	* Macrophyllum *	*M. macrophyllum*	A	LC	Imperiled
	* Mimon *	*M. cozumelae*	A	LC	
	* Phylloderma *	*P. stenops*	A	LC	
	* Tonatia *	*T. bakeri*	A*	LC	
	* Trachops *	*T. coffini*	A*	LC	
	* Vampyrum *	*V. spectrum*	P	NT	Imperiled
** Stenodermatinae **	* Dermanura *	*D. watsoni*	Pr	LC	
	* Enchisthenes *	*E. hartii*	Pr	LC	
	* Phyllops *	*P. falcatus*	P	LC	Critically Imperiled
** Molossidae **	* Cynomops *	*C. mexicanus*	Pr	LC	
	* Eumops *	*E. nanus*	Pr	LC	
** Vespertilionidae **	* Bauerus *	*B. dubiaquercus*	NL	NT	
	* Corynorhinus *	*C. mexicanus*	NL	NT	
	* Euderma *	*E. maculatum*	Pr	LC	
	* Lasionycteris *	*L. noctivagans*	Pr	LC	
	* Myotis *	*M. albescens*	Pr	LC	
		*M. carteri*	Pr	LC	
		*M. evotis*	Pr	LC	
		*M. findleyi*	NL	EN	
		*M. planiceps*	P	EN	Critically Imperiled
		*M. vivesi*	P	VU	Imperiled
	* Perimyotis *	*P. subflavus*	NL	VU	Vulnerable
	* Rhogeessa *	*R. genowaysi*	A	EN	
		*R. mira*	Pr	VU	

The risk category is indicated according to NOM-059-SEMARNAT-2025: Not listed = NL; Subject to Special Protection = Pr; Threatened = A; Endangered = P. The risk categories according to the IUCN are also provided: Least Concern = LC; Near Threatened = NT; Vulnerable = VU; Endangered = EN. The species indicated with an asterisk (*) are listed in SEMARNAT (2025) with their previous taxonomy.

In addition, several species not listed as at risk under NOM-059-SEMARNAT-2025 have been identified as of conservation concern in other assessments. Notably, the study by Adams et al. (2024) highlights species such as *Lichonycteris obscura* Thomas, 1895, *Trinycteris nicefori* (Sanborn, 1949), and *Perimyotis subflavus* (F. Cuvier, 1832) as imperiled or vulnerable, pointing to significant gaps in Mexico’s national evaluation framework.

Finally, several species are marked with an asterisk in the table due to recent taxonomic updates, such as *Gardnerycteris keenani* (Handley, 1960) and *Lophostoma nicaraguae* (Goodwin, 1942), reflecting the need to incorporate advances in systematics to adequately represent the country’s biological diversity.

### ﻿Strategies of bats: migration and hibernation

Bats must adopt different strategies to cope with seasonal food shortages and low winter temperatures in temperate regions: to hibernate or migrate. In Mexico, a total of nine species are currently recognized as migratory, distributed across three families: Molossidae, Phyllostomidae, and Vespertilionidae (Table 5; Laverty et al. 2025; Burke et al. 2019). Regarding hibernation, 14 species have been recorded as hibernators, all belonging exclusively to the family Vespertilionidae (Table 4; Ramos-H et al. 2024). Among these, *Lasiurus cinereus* (Palisot de Beauvois, 1796) is the only species known to combine both migration and hibernation, representing a significant adaptive strategy that warrants further study (Marín et al. 2021; Baerwald et al. 2014; Wieringa et al. 2021). In contrast, for *Nyctinomops macrotis* (Gray, 1839; Mora et al. 2016) and *Myotis planiceps* (Jiménez 1968), there is insufficient evidence to determine whether they migrate or hibernate, respectively, although we suspect that *M. planiceps* is a hibernating species.

**Table 5.** Migratory or hibernating bats in Mexico.

Family	Genus	Species	Migratory	Hibernating
** Molossidae **	* Nyctinomops *	*N. macrotis*	P	
	* Tadarida *	*T. brasiliensis*	*	
** Phyllostomidae **	* Choeronycteris *	*C. mexicana*	*	
	* Leptonycteris *	*L. nivalis*	*	
		*L. yerbabuenae*	*	
** Vespertilionidae **	* Corynorhinus *	*C. leonpaniaguae*		*
		*C. mexicanus*		*
		*C. townsendii*		*
	* Eptesicus *	*E. fuscus*		*
	* Lasionycteris *	*L. noctivagans*	*	
	* Lasiurus *	*L. borealis*	*	
		*L. cinereus*	*	*
		*L. frantzii*	*	
		*L. xantinus*	*	
	* Myotis *	*M. auriculus*		*
		*M. californicus*		*
		*M. ciliolabrum*		*
		*M. occultus*		*
		*M. planiceps*		P
		*M. thysanodes*		*
		*M. velifer*		*
		*M. volans*		*
		*M. yumanensis*		*
	* Perimyotis *	*P. subflavus*		*

An asterisk (*) indicates that the species is a confirmed migrant or hibernating species (Ramos-H et al. 2024). “P” indicates that the species probably migrates or hibernates, but no solid evidence exists to confirm this.

## ﻿Discussion

The recognition of 146 bat species in Mexico reflects not only an increase in the number of known species, but also significant progress in understanding their diversity through integrative taxonomic approaches and expanded sampling efforts in key regions. The application of both molecular and morphological tools has facilitated the description and validation of species such as *Vampyressa villai* and *Corynorhinus leonpaniaguae*, both endemic to Mexico, and has also led to the taxonomic reassessment of species such as *Natalus lanatus*.

Taxonomic revisions affecting 23 bat species represent significant progress in the understanding of Mexico’s biodiversity as reflected in our results. Supported by molecular, morphological, and ecological data, these changes have allowed for a more refined classification, especially within taxonomically challenging groups such as Phyllostomidae and Vespertilionidae.

### ﻿Distribution of richness of bats in Mexico

The geographic distribution of bat species richness in Mexico aligns with well-established biogeographic patterns, with the Mexican Transition Zone (MTZ) serving as a key area in the organization of biodiversity. This region, which marks the boundary between the Nearctic and Neotropical biogeographic provinces, includes major mountain systems such as the Trans-Mexican Volcanic Belt, the Sierra Madre Oriental, the Sierra Madre Occidental, and portions of the Balsas Basin and Sierra Madre del Sur (Morrone 2019). The region’s complex topography, coupled with climatic heterogeneity, gives rise to a wide array of microhabitats that support the coexistence of tropical and temperate species, thus enhancing overall species richness (Ceballos et al. 1998; García et al. 2022).

Recognizing bat species richness patterns is critical for pinpointing conservation priority areas and anticipating the impacts of anthropogenic disturbances. Some bat species, particularly nectarivorous and insectivorous taxa, exhibit narrow ecological ranges that coincide with heavily altered landscapes, thereby heightening their vulnerability (Arriaga-Flores et al. 2018). Consequently, effective conservation of bat diversity in Mexico must consider both biogeographic gradients and the ecological integrity of tropical and transitional ecosystems.

### ﻿Endemism of bats in Mexico

Mexico ranks as the country with the highest number of endemic bat species worldwide, hosting 20 species distributed across four families and 12 genera. This remarkable level of endemism reflects the evolutionary uniqueness of the national chiropteran fauna, particularly within the Mexican Transition Zone (MTZ), where the tropical dry forests of the west and south concentrate a substantial portion of these species. Despite their ecological importance, these areas are increasingly subject to deforestation and land-use change, significantly raising the vulnerability of the species that depend on them.

Several of these endemic species exhibit highly restricted ranges or geographic isolation. For example, *Myotis vivesi* is confined to islands in the Gulf of California and adjacent coastal regions of the Baja California Peninsula and northwestern Mexico. *Myotis planiceps*, on the other hand, is regarded as one of the continental bat species with the smallest distribution in the world, and greatest extinction risk (Arroyo-Cabrales et al. 2005). Notably, its range partially overlaps with that of *Corynorhinus leonpaniaguae*, another recently described endemic species (López-Cuamatzi et al. 2024).

The recognition of *Baeodon* as a genus distinct from *Rhogeessa* increases to two the number of bat genera endemic to Mexico: *Musonycteris* and *Baeodon*. The taxonomic revisions reflected in our study underscore the critical need to reassess and update existing conservation strategies, given that many of these species are classified within risk categories in both the NOM-059-SEMARNAT and the IUCN Red List, reflecting their pronounced ecological vulnerability. Accurate taxonomic delimitation enhances our understanding of biodiversity patterns and facilitates the setting of informed priorities for research, monitoring, and management. Overlooking these developments risks underrepresenting the true number of species at risk and undermining the efficacy of conservation measures.

Globally, the extent of Mexico’s chiropteran endemism stands out even more when compared to other megadiverse nations. For instance, Colombia, home to the second greatest bat diversity worldwide, with 222 species, reports only nine endemic species (Ramírez-Chaves et al. 2024). Likewise, Ecuador lists seven endemic species among its 182 bat species (Tirira et al. 2024). It may be possible that other countries may have more endemic species than Mexico, but for the time being with the available published information, Mexico currently holds the distinction of having the highest number of endemic bat species worldwide.

### ﻿Conservation status

The discrepancies between national and international conservation lists highlight a lack of harmonization that could undermine the effectiveness of bat protection strategies in Mexico. While the newly proposed NOM-059-SEMARNAT-2025 lists 38 species within various risk categories, the IUCN Red List recognizes only 14, with merely five species sharing consistent status across both systems. For example, most of the species classified as “Threatened” under NOM-059-SEMARNAT-2025 are listed as “Least Concern” by the IUCN, with the exception of *Choeronycteris mexicana* (Tschudi, 1844; Near Threatened), *Leptonycteris nivalis* (Saussure, 1860), and *Rhogeessa genowaysi* (both listed as Endangered by the IUCN). Similarly, the recently recorded presence of *Phyllops falcatus* and *Phyllostopmus hastatus* in Mexico, known from only a few or one record, indicate that these are small and likely threatened populations (Aranda-Coello et al. 2025; Rivas-Camo et al. 2020). Both species are classified as least concern by IUCN, while *P. hastatus* is listed as Endangered in neighboring countries such as Guatemala (CONAP 2022), reflecting regional differences in population status and potential threats. As a result, *P. falcatus* is already listed by SEMARNAT (2025) and by Adams et al. (2024) as Endangered, while *P. hastatus* should be considered for inclusion in the next iteration of NOM-059 SEMARNAT list, likely as Endangered. Another example is *Vampyrum spectrum* (Linnaeus, 1758), which is listed as Endangered by SEMARNAT (2025) and considered Near Threatened by the IUCN, despite being classified as Endangered in neighboring countries such as Guatemala (CONAP 2022).

In contrast, five species considered at risk by the IUCN have not been included in NOM-059-SEMARNAT-2025: *Bauerus dubiaquercus* (Van Gelder, 1959) and *Corynorhinus mexicanus* G. M. Allen, 1916 (Near Threatened), *Balantiopteryx io* Thomas, 1904 and *Perimyotis subflavus* (Vulnerable), and *Myotis findleyi* Bogan, 1978 (Endangered). These inconsistencies may be due to differences in assessment criteria, data availability, or the methodological approaches used by each institution. Nonetheless, their existence highlights the urgent need to promote comprehensive and up-to-date evaluations that incorporate the most recent available knowledge, such as that compiled in this article.

In contrast, the case of *Leptonycteris yerbabuenae* stands out, having been recently removed from Mexico’s endangered species list after being recognized as a “recovered” species. This decision reflects the success of coordinated international efforts in conservation, environmental education, monitoring, and ecological restoration. *L. yerbabuenae* thus becomes the first species to be officially delisted due to recovery in both Mexico and the United States, supported by strong evidence of population rebound (Medellín and Torres-Knoop 2015; Adams et al. 2024).

Nonetheless, the delisting of *Leptonycteris yerbabuenae* should not be seen as an endpoint for conservation efforts. As various authors have emphasized, the positive outcomes of these strategies have also benefited other bat species that remain at risk. Moreover, *L. yerbabuenae* plays a critical ecological role as a pollinator of numerous plant species in arid and tropical dry ecosystems, including several *Agave* species of significant ecological, cultural, and economic value. Conserving this species thus entails safeguarding not only its populations, but also the vital ecological processes it helps sustain (Trejo-Salazar et al. 2016).

Additionally, Adams et al. (2024) emphasize that the lack of alignment between countries in recognizing at-risk species represents a significant barrier to effective protection. This issue is exacerbated by substantial knowledge gaps regarding the ecology, distribution, and migratory patterns of many species, which complicate risk assessments and management strategies. Nevertheless, the absence of complete information should not be a justification for inaction; when faced with clear threats, legally recognizing species as at risk is a vital measure to catalyze conservation efforts, as exemplified by the successful case of *Leptonycteris yerbabuenae*.

Overall, the integration of updated taxonomic information and the harmonization between national and international conservation categories are fundamental steps toward more effective and scientifically informed conservation. As one of the world’s megadiverse countries, Mexico faces both the challenge and the opportunity to align its conservation instruments with current scientific knowledge, thereby strengthening its ability to protect its exceptional bat diversity, including many endemic species or those with irreplaceable ecological roles.

### ﻿Strategies of bats: migration and hibernation

We recommend the inclusion of the categories of migratory/hibernating in other lists as these traits can increase the level of endangerment (Adams et al. 2024). A few, like *Lasiurus cinereus*, seem to both migrate and hibernate, but very little is known about their hibernation and much more about their migration (Marín et al. 2021; Baerwald et al. 2014; Wieringa et al. 2021). It is important to promote further research and understanding of these topics.

## ﻿Conclusions

It is clear that Mexico has been a hub of bat studies for decades. Today the country contains 146 bat species, but taxonomy is only catching up and we expect many more species to be discovered in the next decade or so. The geographical features of Mexico, including its very complex topography with many mountain ranges crisscrossing the country from north to south and from east to west, as well as its latitudinal stretch containing the Tropic of Cancer, Mexico has more endemic species of bats and other mammals, than any other country in the world. As far as we know, no other country in the world surpasses the 20 endemic species of bats in Mexico. This is both a great distinction, and also an enormous responsibility; the conservation of those 20 species is the sole obligation of Mexico. Many of these endemic species inhabit the tropical dry forest of the west, others along the Trans-Mexican volcanic belt, and others in the Gulf of California and its islands. Additional endemic species can be found in the northeastern Sierra Madre Oriental. Evidently, much more research is still needed for the taxonomy of Mexican bats to be finally understood in its entirety. Some taxonomic gaps that warrant urgent attention are the genera *Myotis*, *Eptesicus*, *Neoeptesicus*, *Rhogeessa*, *Eumops*, and even groups like *Micronycteris*, *Perimyotis*, and *Molossus*. Geographically, additional taxonomic work on bats is urgently needed in the rainforests of Chiapas and Oaxaca, and also in the montane forests of the Trans-Mexican volcanic belt.

In terms of extinction risk, focusing on those categories that indeed represent risk of extinction, that is A and P in the Mexican legislation and Vu, En, and Cr according to IUCN (see Table 3), only five species: *L. nivalis* (A, En), *M. harrisoni* (P, Vu), *M. planiceps* (P, En), *M. vivesi* (P, Vu), *R. genowaysi* (A, En) have somewhat coincidental classification rankings. This is surprising and concerning, given that 38 species are listed in the Mexican legislation, although 17 of those are under Special Protection (Pr), a category that does not necessarily represent extinction risk (SEMARNAT 2025), and of the 14 species listed under the IUCN categories of NT, Vu, En, and Cr, only 9 are included in the three categories that IUCN considers to be true risk of extinction (Vu, En, Cr). But this limited coincidence raises concerns about the equivalence or objectivity shared between these two systems. Our analysis serves as a basis to continue promoting converging, more robust classification in the two systems. For one, homologation among the three systems used here, IUCN, the Mexican legislation, and the expert elicitation (Adams et al. 2024) would be desirable, and an analysis of which species in need of conservation efforts require attention is urgent.

Bat species lists at the country level are an important source of information that can help the decision-making process and conservation policy. For example, to set biodiversity monitoring practices and conservation priorities within the country, to conduct assessments of ecological importance and ecosystem services, to guide legislation and environmental impact assessment, and these lists also are the first step for neighboring countries to identify and plan collaborative conservation programs aimed at protecting shared species, or even individuals when those species migrate from one country to another. These lists also should be revisited every few years to keep them accurate and up to date.

## ﻿Additional information

### Use of AI

No use of AI was reported.

### Author ORCIDs

Ameyalli Marín-Ventura https://orcid.org/0009-0002-6876-3330

Liliana Rosas-Durán https://orcid.org/0009-0000-1465-6594

Cárol Sierra-Durán https://orcid.org/0000-0002-7956-6204

Joaquín Arroyo-Cabrales https://orcid.org/0000-0002-9095-489X

### Data availability

All of the data that support the findings of this study are available in the main text.
